# Effect of Adding Personalized Instant Messaging Apps to a Brief Smoking Cessation Model in Community Smokers in Hong Kong: Pragmatic Randomized Clinical Trial

**DOI:** 10.2196/44973

**Published:** 2024-05-13

**Authors:** Yongda Socrates Wu, Yee Tak Derek Cheung, Jay Jung Jae Lee, Carlos King Ho Wong, Sai Yin Ho, William Ho Cheung Li, Ying Yao, Tai Hing Lam, Man Ping Wang

**Affiliations:** 1 School of Nursing The University of Hong Kong Hong Kong China (Hong Kong); 2 Children's Hospital of Eastern Ontario Research Institute Ottawa, ON Canada; 3 Department of Family Medicine and Primary Care The University of Hong Kong Hong Kong China (Hong Kong); 4 School of Public Health The University of Hong Kong Hong Kong China (Hong Kong); 5 Nethersole School of Nursing The Chinese University of Hong Kong Hong Kong China (Hong Kong)

**Keywords:** instant messaging, text messaging, chatting, smoking cessation, COVID-19, community smoker

## Abstract

**Background:**

While text messaging has proven effective for smoking cessation (SC), engagement in the intervention remains suboptimal.

**Objective:**

This study aims to evaluate whether using more interactive and adaptive instant messaging (IM) apps on smartphones, which enable personalization and chatting with SC advisors, can enhance SC outcomes beyond the provision of brief SC advice and active referral (AR) to SC services.

**Methods:**

From December 2018 to November 2019, we proactively recruited 700 adult Chinese daily cigarette users in Hong Kong. Participants were randomized in a 1:1 ratio. At baseline, all participants received face-to-face brief advice on SC. Additionally, they were introduced to local SC services and assisted in selecting one. The intervention group received an additional 26 personalized regular messages and access to interactive chatting through IM apps for 3 months. The regular messages aimed to enhance self-efficacy, social support, and behavioral capacity for quitting, as well as to clarify outcome expectations related to cessation. We developed 3 sets of messages tailored to the planned quit date (within 30 days, 60 days, and undecided). Participants in the intervention group could initiate chatting with SC advisors on IM themselves or through prompts from regular messages or proactive inquiries from SC advisors. The control group received 26 SMS text messages focusing on general health. The primary outcomes were smoking abstinence validated by carbon monoxide levels of <4 parts per million at 6 and 12 months after the start of the intervention.

**Results:**

Of the participants, 505/700 (72.1%) were male, and 450/648 (69.4%) were aged 40 or above. Planning to quit within 30 days was reported by 500/648 (77.2%) participants, with fewer intervention group members (124/332, 37.3%) reporting previous quit attempts compared with the control group (152/335, 45.4%; *P*=.04). At the 6- and 12-month follow-ups (with retention rates of 456/700, 65.1%, and 446/700, 63.7%, respectively), validated abstinence rates were comparable between the intervention (14/350, 4.0%, and 19/350, 5.4%) and control (11/350, 3.1% and 21/350, 6.0%) groups. Compared with the control group, the intervention group reported greater utilization of SC services at 12 months (RR 1.26, 95% CI 1.01-1.56). Within the intervention group, engaging in chat sessions with SC advisors predicted better validated abstinence at 6 months (RR 3.29, 95% CI 1.13-9.63) and any use of SC services (RR 1.66, 95% CI 1.14-2.43 at 6 months; RR 1.67, 95% CI 1.26-2.23 at 12 months).

**Conclusions:**

An IM-based intervention, providing support and assistance alongside brief SC advice and AR, did not yield further increases in quitting rates but did encourage the utilization of SC services. Future research could explore whether enhanced SC service utilization leads to improved long-term SC outcomes.

**Trial Registration:**

ClinicalTrials.gov NCT03800719; https://clinicaltrials.gov/ct2/show/NCT03800719

## Introduction

Smoking cessation (SC) can effectively alleviate the burden of diseases caused by tobacco use [1]. SMS text messaging has proven to be effective for SC [2,3]. The effectiveness of SMS text messaging for SC primarily lies in providing psychosocial support [4]. Exploring more interactive and adaptable platforms, such as instant messaging (IM) apps on smartphones that offer personalization and the ability to chat with SC advisors, might offer enhanced support, potentially leading to increased success in SC efforts. The World Health Organization has also embraced platforms such as WhatsApp (Meta Platforms, Inc.) and Facebook Messenger (Meta Platforms, Inc.) to encourage quitting [5], although the evidence regarding their effectiveness remains uncertain. Our literature search (see Multimedia Appendix 1) yielded only 6 relevant randomized controlled trials (RCTs), with full results provided in Multimedia Appendix 2, highlighting the lack of conclusive evidence regarding the effectiveness of IM-based interventions for SC [6-11].

Smartphone usage is widespread in Hong Kong [12], the most Westernized city in China, where WhatsApp and WeChat (Tencent Holdings Ltd.) are the predominant IM apps. In our previous RCT involving community smokers, we offered chat-based support through IM alongside brief SC advice delivered face-to-face [13]. The combined intervention resulted in a 70% increase in the odds of validated abstinence compared with receiving only brief SC advice. However, the level of engagement in chatting was low (16.8%), a trend commonly observed in trials using digital interventions for diverse health concerns [14,15]. Previous studies have indicated that engagement levels can predict successful quitting outcomes [13,15], echoing similar findings in digital interventions aimed at addressing various health issues [14]. These results emphasize the importance of investigating strategies to enhance engagement, including the delivery of multimedia messages and tailoring interventions to individual quitting-related characteristics [16].

SC services provide effective interventions, including nicotine replacement therapies (NRTs), medications such as varenicline and bupropion, and behavioral support [17]. However, these services are underutilized worldwide, as highlighted by the Global Adult Tobacco Survey, which revealed that only a quarter of smokers who attempted to quit in the past 12 months accessed SC services [18]. Similarly, only 4.9% of smokers in Hong Kong had previously used SC services or medications, despite the fact that these services are largely available free of charge or at a nominal fee (HK $50 [US $6.40]). Among those who had not previously used these services or medications, a staggering 94.0% expressed reluctance to consider trying them [19,20]. Over a quarter of local smokers in Hong Kong were classified as hardcore smokers, having never attempted to quit and expressing no desire to do so [21]. Consequently, the prevalence of smoking in Hong Kong has reached a standstill in recent years, with rates at 9.8% (daily) and 0.6% (nondaily) as of 2021 [20]. The majority of smokers were male (83.1%) and aged 40 years or older, with 25.1% in the 40-49-year age group, 23.0% in the 50-59-year age group, and 27.6% aged 60 years or older.

Proactive strategies have proven effective in boosting the utilization of SC services among smokers recruited from clinical or primary care settings, as demonstrated in numerous studies across the United States [22-25], the United Kingdom [26,27], and Australia [28]. Despite its effectiveness, active referral (AR), which involves passing smokers’ contact information to SC service providers, resulted in only 29.1% of community smokers using SC services during the study period, albeit leading to increased uptake of SC services and higher rates of smoking abstinence [29]. The primary reasons cited for not using SC services were a busy schedule, timing conflicts, and lack of interest [29]. Subsequent interventions aimed at enhancing AR strategies, such as on-site referral (where service providers are contacted on behalf of smokers at baseline) [30] and AR coupled with a small financial incentive for SC service utilization [31], demonstrated increased rates of quitting compared with brief SC advice alone. However, only the on-site referral approach showed a slight improvement in SC service utilization, with no comparison made to a trial arm offering AR alone.

This RCT examined a novel IM-based intervention that incorporated personalized psychosocial support and guidance for utilizing SC services, in addition to brief SC advice and AR, among smokers proactively recruited from communities across Hong Kong. Our hypothesis posited that tailored messages could enhance engagement with the IM-based intervention, thereby offering increased support for SC efforts. By enhancing assistance for utilizing SC services alongside personalized support through IM, the intervention has the potential to bolster both SC service utilization and smoking abstinence rates. The findings from this study will offer valuable insights into strategies for enhancing SC support via IM platforms and encouraging the uptake of SC services. If proven effective, this intervention model could serve as a valuable reference for promoting SC in countries or regions where IM is widely used and SC services are readily accessible.

## Methods

### Study Design

This study was a parallel 2-arm, pragmatic RCT conducted within communities across Hong Kong. The protocol has been registered with ClinicalTrials.gov (NCT03800719) and is accessible as Multimedia Appendix 3. We adhered to the CONSORT (Consolidated Standards of Reporting Trials) guidelines for pragmatic trials (Multimedia Appendix 4) [32].

### Ethics Approval

Ethical approval was obtained from the Institutional Review Board of the University of Hong Kong/Hospital Authority Hong Kong West Cluster (UW 18-172). Written informed consent was obtained from all participants, and all data and results presented in this study were deidentified.

### Recruitment and Participants

Recruitment occurred at community smoking hotspots, areas frequented by many smokers for smoking purposes, including locations near shopping centers, housing estates, and metro stations [33]. Adult daily cigarette users were recruited between December 15, 2018, and November 7, 2019. SC advisors, who were university students trained in a half-day workshop and supervised by research staff on-site, proactively approached smokers using a “foot-in-the-door” technique [33,34]. Smokers were initially queried about their smoking history and habits, including their daily cigarette consumption, age at initiation of smoking, and any previous attempts to quit, including the utilization of SC services. Smokers who expressed a willingness to engage were briefly advised to consider quitting or reducing smoking, and those showing interest were extended an invitation to participate in this RCT. To be eligible for participation, individuals had to be daily cigarette users within the past 3 months, as verified by an exhaled carbon monoxide (CO) level of 4 parts per million (ppm) or above, measured at smoking hotspots during recruitment. Additionally, they needed to be Hong Kong residents aged 18 or older, proficient in Chinese, and in possession of a smartphone equipped with an IM app for receiving intervention messages and follow-up. Exclusion criteria encompassed individuals who (1) had psychiatric or psychological disorders diagnosed by a physician or were under treatment for such conditions, (2) were on regular psychotropic medications, or (3) were using SC medications or services.

### Randomization and Blinding

Participants were randomly assigned in a 1:1 ratio using sequentially numbered, opaque, sealed envelopes (SNOSEs). An SC advisor would open 1 SNOSE promptly after receiving written consent from participants. The SNOSEs were prepared by an investigator (YSW) who was not involved in recruitment. They were generated via computerized allocation sequences with block sizes of 4, 8, and 12 in random order. The intervention was administered to 1 smoker at a time to prevent contamination. Following the conclusion of the current intervention, a 5-minute interval was observed to allow the recruited smoker to depart before proceeding to recruit a new smoker at the same smoking hotspot. Allocation concealment was maintained; however, blinding of the SC advisors and participants was not feasible. Nevertheless, all outcome assessors and statistical analysts remained blinded until the primary analyses were concluded.

### Interventions

Upon completing the baseline questionnaire, all participants were provided with brief SC advice, typically lasting 5-10 minutes, delivered face-to-face. This advice was guided by the AWARD (Ask, Warn, Advise, Refer, and Do-it-again) model, which was designed and tested in our previous trials [13,29,35,36]. The AWARD model consists of the following components: *Ask* about smoking history; *Warn* about the high risk of smoking using a health warning leaflet; *Advise* smokers to quit as soon as possible and adhere to the chosen quit date; *Refer* smokers to SC services; and *Do* it again. The AWARD model was adapted and simplified from the 5As (Ask, Advise, Assess, Assist, and Arrange) specifically for implementation in the community setting [37]. Additionally, participants were proactively directed to local SC services. They were briefed about the available SC services in Hong Kong and aided in selecting one based on their preferences regarding service offerings, such as available treatments, operational hours, and locations. The contact details of participants who provided written consent for referral were promptly forwarded to their selected service providers within the following day. Subsequently, the service providers reached out to the referred participants to arrange phone or face-to-face counseling sessions or consultations, typically scheduled within 1-2 weeks.

In addition to the standard intervention, participants in the intervention group received an IM-based intervention using platforms such as WhatsApp or WeChat. This intervention involved receiving personalized messages at regular intervals (refer to Multimedia Appendix 5) and engaging in interactive chatting for 3 months following the initiation of the intervention. The content of the regular messages was guided by the principles of the social cognitive theory, which has been used in previous SC interventions utilizing text messages [38,39]. The IM-based intervention aimed to enhance participants’ self-efficacy, social support, and behavioral capacity for quitting smoking, while also clarifying the expectations associated with quitting. Practical skills and emotional support were provided to assist participants in managing cravings, withdrawal symptoms, situational triggers, lapses, and relapses. These components were designed based on standard information from the SC kit provided by the Department of Health, Hong Kong SAR [40], and informed by evidence from our previous smoking relapse prevention trial [6]. We devised 3 sets of messages (sent through IM, with some also including pictures and videos) tailored to the planned quit date, categorized as within 30 days, within 60 days, and undecided, in alignment with the transtheoretical model (TTM) of change [41]. Following the steps recommended by Abroms et al [42] for message design, we consulted experienced SC counselors from local service providers to refine the messages before finalization. Additionally, messages were personalized based on participants’ sociodemographic characteristics, baseline smoking habits, and updated smoking status obtained during interactive messaging conversations. The primary objective was to boost engagement, defined as active participation in conversations with SC advisors on IM platforms. A total of 26 messages were sent, comprising various formats such as texts, pictures, and videos, aimed at enhancing engagement. The message schedule involved sending messages once daily during the week encompassing the quit date, followed by 3 times a week for 4 weeks (2 weeks before and after the quit date), and once a week for 7 weeks. In instances where a quit date was not established, daily messages were initiated immediately. Furthermore, the frequency and schedule of messages were adjusted based on participants’ preferences as conveyed during IM conversations. Participants had the autonomy to initiate text or voice chats with SC advisors on IM platforms, or these interactions could be prompted by regular messages or proactive inquiries from SC advisors. For instance, advisors might inquire about the current progress of quitting or the utilization of SC services. Specifically, SC advisors reminded participants to utilize the chosen service and provided assistance in addressing any issues encountered in utilizing these services, such as missed appointments or inquiries regarding the location and contact information of service providers. All conversations were meticulously recorded, with 5% randomly selected for review by experienced SC counselors from the research team.

Participants assigned to the control group received 26 messages (refer to Multimedia Appendix 6) focusing on general health information and reminders for follow-up appointments over the same 3-month period. These were delivered as SMS text messages at a frequency comparable to that of the intervention group.

### Outcomes

All participants underwent telephone follow-ups at 3, 6, and 12 months after the initiation of the intervention to evaluate their smoking status. A HK $50 (US $6.40) coupon was provided as compensation for each successful follow-up to acknowledge their time and participation. The primary outcome measure was validated abstinence, defined as an exhaled CO level of less than 4 parts per million (ppm), assessed at 6 and 12 months after the initiation of the intervention. Participants who reported complete abstinence (not even a single puff) in the past 7 days at the 6- and 12-month follow-ups were invited to undergo an exhaled CO test using a piCO Smokerlyzer (Bedfont Scientific), facilitated by research staff. These tests were conducted at outdoor community sites that were convenient for the participants. To acknowledge their time and cover travel expenses, participants received a small cash incentive of HK $300 (US $38.40). It is worth noting that previous trials involving community smokers found that this incentive had no impact on quitting outcomes [43]. The main secondary outcomes were assessed at 6 and 12 months, including self-reported 7-day point prevalence abstinence (PPA), continuous abstinence for 24 weeks, reduction of smoking by at least 50% compared with baseline daily cigarette consumption, any quit attempt (abstinence for 24 hours or longer), and utilization of SC services after intervention initiation. The self-reported utilization of SC services, including even a single session, was validated by cross-referencing with records from service providers. Additionally, participants in the intervention group reported whether they had engaged in chat sessions with SC advisors through IM during the 3-month intervention period, which was verified using chat records. To evaluate the quality of life, the validated EQ-5D-5L was administered at baseline and at the 12-month follow-up [44]. As part of the process evaluation for the IM-based intervention, all participants were requested to provide ratings at the 6-month mark regarding the helpfulness of messages, which also included chatting with participants in the intervention group. Ratings were provided on a scale from 0 (not at all helpful) to 10 (most helpful) for the following aspects: (1) increasing quitting motivation, (2) increasing quitting confidence, and (3) overall helpfulness.

### Sample Size

The sample size was determined using G-Power (Universität Düsseldorf). As no previous studies had reported the effect of IM messages and chatting in addition to brief advice and AR, calculations were based on the following quit rates: (1) a 10.7% biochemically validated quit rate at 6 months, as reported in a previous trial evaluating SMS text messaging–based interventions [45]; and (2) our previous trials, where only brief advice was provided in the control group, observed a 5.0% validated quit rate at 6 months [29,35]. Assuming a type I error of 0.05 and a power of 0.8, as observed in our previous trials involving community smokers, a total of 696 participants would be required (with 348 participants in each group) to detect a significant difference between the intervention and control groups.

### Statistical Analysis

We applied the intention-to-treat approach in the primary analysis, where missing outcome values were imputed with the baseline values. Poisson working models with a log-link were used to assess the intervention effect on primary outcomes, yielding risk ratios (RRs) [46]. Similar analytical methods were applied to conduct prespecified secondary analyses. In our first step, we replicated the primary analysis while adjusting for imbalanced baseline characteristics. Subsequently, subgroup analyses were conducted based on intention to quit, specifically focusing on participants with a planned quit date within 30 days at baseline [41]. Two post hoc analyses have been performed: one was to investigate whether the intervention effects on outcomes at 3 months were consistent with those observed at the subsequent 2 follow-ups and the other was to examine the association between engagement, defined as chatting with SC advisors, and cessation outcomes among participants in the intervention group.

Two prespecified sensitivity analyses were conducted: (1) complete case analyses were conducted, excluding participants with missing outcome data and (2) missing data were handled using multiple imputations by chained equations [47]. The imputation model incorporated outcomes, study group assignment, sociodemographic characteristics (eg, sex, age, highest educational attainment, and monthly household income), and baseline smoking-related characteristics (including daily cigarette consumption, time to the first cigarette after waking, previous quit attempts, and intention to quit). Fifty imputed data sets were generated, and regression results were aggregated according to Rubin’s rule [48]. Responses to the EQ-5D-5L questionnaire were transformed using the standard Hong Kong value set, which ranged from –0.865 (indicating the worst quality of life) to 1 (indicating the best quality of life). The participants’ quality of life was assessed using the area under the receiver operating characteristic curve approach [44]. A 2-sided *P* value of less than .05 was considered statistically significant. All statistical analyses were performed using Stata version 15.1 (StataCorp). Additionally, individual interviews were conducted with participants to gain insights into their perceptions and experiences regarding the IM-based intervention. The findings from these interviews will be presented separately.

## Results

### Characteristics of Participants

**Figure 1:**
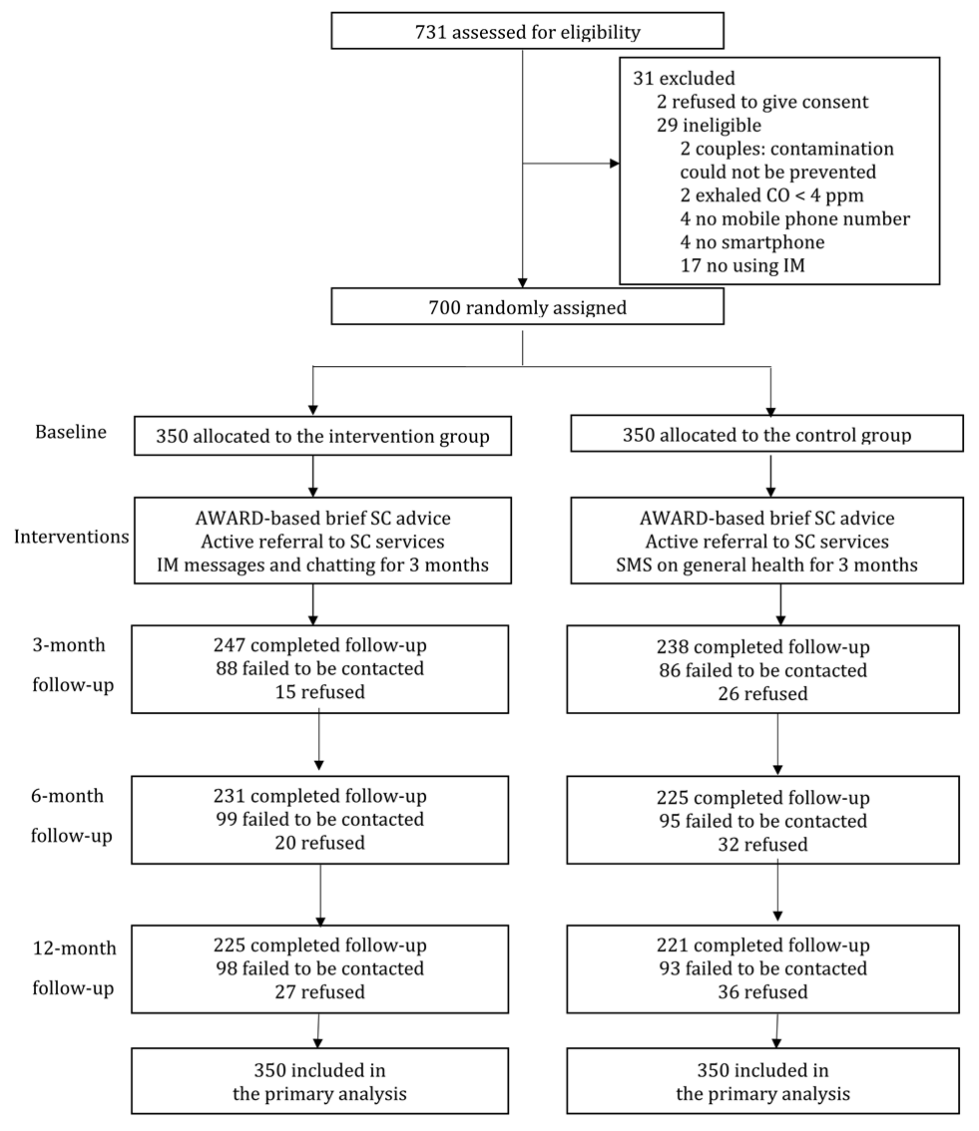
The CONSORT (Consolidated Standards of Reporting Trials) flow diagram. AWARD: Ask, Warn, Advise, Refer, and Do-it-again; CO: carbon monoxide; IM: instant messaging; SC: smoking cessation.

Of the 731 smokers initially screened, 700 were found eligible and provided consent to participate in the RCT. A CONSORT flow diagram illustrating the participant flow is provided in Figure 1. Half of the participants (n=350) were randomly assigned to the intervention group, while the remaining half were allocated to the control group. Table 1 presents the baseline sociodemographic and smoking-related characteristics of the participants. It indicates that these characteristics were generally similar between the 2 groups. In total, 505/700 (72.1%) participants were male, with 450/648 (69.4%) aged 40 years or older, and 425/615 (69.1%) having attained secondary education. Additionally, 288/660 (43.6%) reported smoking their first cigarette within 5 minutes after waking, while 385/695 (55.4%) consumed more than 10 cigarettes daily. Notably, 500/648 (77.2%) expressed intentions to quit smoking within 30 days. A comparison between the intervention and control groups revealed that a lower proportion of participants in the intervention group had made previous quit attempts compared with those in the control group (124/332, 37.3%, vs 152/335, 45.4%; *P*=.04). Nearly all participants in both the intervention (331/350, 94.6%) and control (333/350, 95.1%) groups opted for a SC service at baseline. The overall retention rates at follow-up were 69.3% (485/700) at 3 months, 65.1% (456/700) at 6 months, and 63.7% (446/700) at 12 months. Importantly, these retention rates were comparable between the 2 groups at all follow-up time points (*P*=.46-.75).

**Table 1:** Sociodemographic and smoking-related characteristics, and referral to smoking cessation services at baseline (N=700).

Characteristics			Intervention^a^ (n=350)		Control^a^ (n=350)		*P* value^b^
**Sex, n/N (%)**							.35
	Male	247/350 (70.6)		258/350 (73.7)			
	Female	103/350 (29.4)		92/350 (26.3)			
**Age (years)**							.80
	18-29, n/N (%)	40/322 (12.4)		44/326 (13.5)			
	30-39, n/N (%)	52/322 (16.1)		62/326 (19.0)			
	40-49, n/N (%)	80/322 (24.8)		77/326 (23.6)			
	50-59, n/N (%)	82/322 (25.5)		73/326 (22.4)			
	60 or above, n/N (%)	68/322 (21.1)		70/326 (21.5)			
	Missing, n	28		24			
**Highest educational attainment**							.80
	Primary or below, n/N (%)	41/305 (13.4)		42/310 (13.5)			
	Secondary, n/N (%)	214/305 (70.2)		211/310 (68.1)			
	Tertiary, n/N (%)	50/305 (16.4)		57/310 (18.4)			
	Missing, n	45		40			
**Marital status**							.18
	Single, n/N (%)	85/315 (27.0)		85/314 (27.1)			
	Married/cohabited, n/N (%)	216/315 (68.6)		204/314 (65.0)			
	Divorced/widow, n/N (%)	14/315 (4.4)		25/314 (8.0)			
	Missing, n	35		36			
**Children living together**							.75
	No, n/N (%)	204/297 (68.7)		218/312 (69.9)			
	Yes, n/N (%)	93/297 (31.3)		94/312 (30.1)			
	Missing, n	53		38			
**Housing**							.09
	Rent, n/N (%)	168/310 (54.2)		196/316 (62.0)			
	Owned, n/N (%)	137/310 (44.2)		113/316 (35.8)			
	Others, n/N (%)	5/310 (1.6)		7/316 (2.2)			
	Missing, n	40		34			
**Employment status^c^**							.38
	Economically active, n/N (%)	213/319 (66.8)		217/310 (70.0)			
	Economically inactive, n/N (%)	106/319 (33.2)		93/310 (30.0)			
	Missing, n	31		40			
**Monthly household income (HK $^d^)**							.78
	Unstable/<19,999, n/N (%)	125/295 (42.4)		137/303 (45.2)			
	20,000-29,999, n/N (%)	88/295 (29.8)		85/303 (28.1)			
	30,000 or above, n/N (%)	82/295 (27.8)		81/303 (26.7)			
	Missing, n	55		47			
**Time to the first cigarette after waking**							.72
	After 60 minutes, n/N (%)	51/330 (15.5)		51/330 (15.5)			
	31-60 minutes, n/N (%)	42/330 (12.7)		33/330 (10.0)			
	6-30 minutes, n/N (%)	94/330 (28.5)		101/330 (30.6)			
	Within 5 minutes, n/N (%)	143/330 (43.3)		145/330 (43.9)			
	Missing, n	20		20			
**Daily cigarette consumption**							.70
	1-10, n/N (%)	161/349 (46.1)		149/346 (43.1)			
	11-20, n/N (%)	147/349 (42.1)		150/346 (43.4)			
	21-30, n/N (%)	24/349 (6.9)		31/346 (9.0)			
	31 or above, n/N (%)	17/349 (4.9)		16/346 (4.6)			
	Missing, n	1		4			
The Heaviness of Smoking Index, median (IQR)^e^			3 (2-4)		3 (2-4)		.48
**Previous quit attempts**							.04
	No attempt, n/N (%)	208/332 (62.7)		183/335 (54.6)			
	Had attempts, n/N (%)	124/332 (37.3)		152/335 (45.4)			
	Missing, n	18		15			
**Intention to quit**							.41
	Within 7 days, n/N (%)	145/325 (44.6)		135/323 (41.8)			
	Within 30 days, n/N (%)	113/325 (34.8)		107/323 (33.1)			
	Within 60 days, n/N (%)	21/325 (6.5)		32/323 (9.9)			
	Undecided, n/N (%)	46/325 (14.2)		49/323 (15.2)			
	Missing, n	25		27			
**Perceptions of quitting (score: 0-10)^f^**							
	Perceived importance of quitting (n=621), median (IQR)	7 (5-10)		7 (5-10)		.71	
	Perceived difficulty of quitting (n=621), median (IQR)	7 (5-9)		7 (5-9)		.82	
	Perceived confidence of quitting (n=620), median (IQR)	5 (5-6)		5 (5-6)		.78	
Referred to smoking cessation services, n/N (%)			331/350 (94.6)		333/350 (95.1)		.73

^a^Number of missing values excluded from the calculations of the column percentages

^b^*P* values from chi-square tests and Wilcoxon rank-sum tests excluding missing values. Between-group differences were due to randomization (chance); *P* values are for references only.

^c^Being an employer, employee, or self-employed was regarded as economically active; being a student, housekeeper, retired, or unemployed was regarded as economically inactive.

^d^US $1=HK $7.8.

^e^Scored 0-6, with the higher score indicating a higher level of dependence.

^f^Scored 0-10, with the higher score indicating stronger perceptions.

### Primary and Sensitivity Analyses

Table 2 indicates that the validated abstinence rates between the intervention and control groups at 6 months (14/350, 4.0%, vs 11/350, 3.1%) and 12 months (19/350, 5.4%, vs 21/350, 6.0%) did not exhibit a significant difference (6-month RR 1.27, 95% CI 0.59-2.77 and 12-month RR 0.90, 95% CI 0.50-1.65). Both groups demonstrated similar rates of self-reported 7-day PPA, smoking reduction by 50% (including self-reported quitters), and any quit attempt at 6 and 12 months. In the intervention group, self-reported 24-week continuous abstinence was notably higher at 6 months (11/350, 3.1%, vs 3/350, 0.9%; RR 3.67, 95% CI 1.03-13.04), while any use of SC services was significantly higher at 12 months (123/350, 35.1%, vs 98/350, 28.0%; RR 1.26, 95% CI 1.01-1.56). Analyses adjusting for previous quit attempts at baseline yielded similar results to those presented earlier. Sensitivity analyses conducted based on complete cases and multiple imputation methods also produced consistent findings regarding abstinence outcomes, as demonstrated in Table S2 in Multimedia Appendix 7. Moreover, outcomes did not exhibit significant differences between the 2 groups at the 3-month mark, as illustrated in Table S3 in Multimedia Appendix 7. For instance, self-reported 7-day PPA was comparable between the intervention and control groups (35/350, 10.0%, vs 25/350, 7.1%; RR 1.51, 95% CI 0.93-2.45).

**Table 2:** Primary and secondary outcomes in the intervention and control groups (N=700).

Outcomes					Intervention (n=350), n (%)		Control (n=350), n (%)		Crude risk ratio (95% CI)		Adjusted risk ratio^a^ (95% CI)
**Primary outcomes**											
	**Validated abstinence**										
			6 months	14 (4.0)		11 (3.1)		1.27 (0.59-2.77)		1.31 (0.60-2.85)	
			12 months	19 (5.4)		21 (6.0)		0.90 (0.50-1.65)		0.95 (0.52-1.75)	
**Secondary outcomes**											
	**Self-reported 7-day** **point prevalence abstinence**										
		6 months		32 (9.1)		29 (8.3)		1.10 (0.68-1.78)		1.17 (0.73-1.88)	
		12 months		46 (13.1)		50 (14.3)		0.92 (0.63-1.33)		0.97 (0.67-1.41)	
	**Self-reported 24-week continuous abstinence**										
		6 months		11 (3.1)		3 (0.9)		3.67 (1.03-13.04)^b^		3.74 (1.07-13.12)^b^	
		12 months		22 (6.3)		21 (6.0)		1.05 (0.59-1.87)		1.15 (0.66-2.02)	
	**Smoking reduction by at least 50% of baseline^c^**										
		6 months		100 (28.6)		81 (23.1)		1.23 (0.96-1.59)		1.26 (0.97-1.62)	
		12 months		111 (31.7)		122 (34.9)		0.91 (0.74-1.12)		0.92 (0.74-1.13)	
	**Any quit attempt since intervention initiation**										
		6 months		49 (14.0)		36 (10.3)		1.36 (0.91-2.04)		1.45 (0.97-2.16)	
		12 months		72 (20.6)		63 (18.0)		1.14 (0.84-1.55)		1.21 (0.89-1.63)	
	**Any use of** **smoking cessation** **service since intervention initiation**										
		6 months		90 (25.7)		84 (24.0)		1.07 (0.83-1.39)		1.06 (0.82-1.38)	
		12 months		123 (35.1)		98 (28.0)		1.26 (1.01-1.56)^b^		1.26 (1.01-1.57)^b^	

^a^Adjusted for previous quit attempts.

^b^*P*<.05.

^c^Self-reported quitters included.

### Subgroup and Post Hoc Analyses

Table 3 illustrates that among smokers without an intention to quit (ie, those with a planned quit date beyond 30 days or undecided), the intervention group tended to exhibit worse quitting outcomes, although these differences were not statistically significant. Conversely, among participants who had expressed an intention to quit, the intervention group generally demonstrated better quitting outcomes. Specifically, significant results were observed for any quit attempt at 6 months (adjusted RR [ARR] 1.74, 95% CI 1.10-2.76) and any use of SC services at 12 months (ARR 1.31, 95% CI 1.03-1.67). Table S1 in Multimedia Appendix 7 reveals that 74/350 (21.1%) participants in the intervention group engaged in chats with SC advisors during the intervention period. Interestingly, a higher proportion of these participants reported having children living with them and expressed an intention to quit smoking (*P*=.05 in all cases). Table S4 in Multimedia Appendix 7 demonstrates that among the intervention group participants who engaged in chats with SC advisors, there were notable improvements in various quitting outcomes. Specifically, these participants exhibited significantly higher rates of validated abstinence at 6 months (ARR 3.29, 95% CI 1.13-9.63), smoking reduction by at least 50% (ARR 1.75, 95% CI 1.21-2.55 at 6 months; ARR 1.92, 95% CI 1.36-2.71 at 12 months), and any use of SC services (ARR 1.66, 95% CI 1.14-2.43 at 6 months; ARR 1.67, 95% CI 1.26-2.23 at 12 months). Table S5 in Multimedia Appendix 7 indicates that participants in the intervention group who engaged in chats with SC advisors rated the intervention as more helpful compared with those who did not engage in chats or participants in the control group.

**Table 3:** Subgroup analyses by intention to quit (planned to quit in 30 days) at baseline (N=648, after excluding 52 participants not reporting intention to quit at baseline).

			No intention to quit (n=148)									Had intention to quit (n=500)									
			Intervention (n=67), n (%)		Control (n=81), n (%)		Crude risk ratio (95% CI)		Adjusted risk ratio^a^ (95% CI)		Intervention (n=258), n (%)			Control (n=242), n (%)		Crude risk ratio (95% CI)		Adjusted risk ratio^a^ (95% CI)			
**Validated abstinence**																					
	6 months	1 (1.5)		3 (3.7)		0.40 (0.04-3.81)		0.40 (0.05-3.61)		12 (4.7)			8 (3.3)		1.41 (0.58-3.39)		1.45 (0.60-3.51)				
	12 months	4 (6.0)		4 (4.9)		1.21 (0.31-4.67)		1.29 (0.35-4.77)		15 (5.8)			17 (7.0)		0.83 (0.42-1.62)		0.87 (0.44-1.71)				
**Self-reported 7-day** **point prevalence abstinence**																					
	6 months	5 (7.5)		9 (11.1)		0.67 (0.24-1.92)		0.66 (0.24-1.86)		26 (10.1)			19 (7.9)		1.28 (0.73-2.26)		1.39 (0.79-2.42)				
	12 months	8 (11.9)		8 (9.9)		1.21 (0.48-3.06)		1.22 (0.48-3.05)		36 (14.0)			39 (16.1)		0.87 (0.57-1.32)		0.93 (0.61-1.41)				
**Self-reported 24-week continuous abstinence^b^**																					
	6 months	2 (3.0)		0 (0.0)		NA		NA		9 (3.5)			3 (1.2)		2.81 (0.77-10.29)		2.97 (0.82-10.76)				
	12 months	3 (4.5)		4 (4.9)		0.91 (0.31-3.93)		0.99 (0.24-4.12)		18 (7.0)			15 (6.2)		1.13 (0.58-2.18)		1.23 (0.64-2.34)				
**Smoking reduction by at least 50% of baseline^c^**																					
	6 months	18 (26.9)		20 (24.7)		1.09 (0.63-1.89)		1.07 (0.62-1.84)		76 (29.5)			57 (23.6)		1.25 (0.93-1.68)		1.29 (0.96-1.73)				
	12 months	15 (22.4)		24 (29.6)		0.76 (0.43-1.32)		0.76 (0.43-1.33)		86 (33.3)			89 (36.8)		0.91 (0.71-1.15)		0.92 (0.72-1.17)				
**Any quit attempt since intervention initiation**																					
	6 months	7 (10.4)		11 (13.6)		0.77 (0.31-1.88)		0.77 (0.32-1.89)		41 (15.9)			24 (9.9)		1.60 (1.00-2.57)		1.74 (1.10-2.76)^d^				
	12 months	11 (16.4)		15 (18.5)		0.89 (0.44-1.80)		0.89 (0.44-1.81)		59 (22.9)			45 (18.6)		1.23 (0.87-1.74)		1.32 (0.94-1.85)				
**Any use of** **smoking cessation** **service since intervention initiation**																					
	6 months	7 (10.4)		12 (14.8)		0.71 (0.29-1.70)		0.70 (0.29-1.69)		79 (30.6)			66 (27.3)		1.12 (0.85-1.48)		1.12 (0.85-1.47)				
	12 months	11 (16.4)		15 (18.5)		0.89 (0.44-1.80)		0.88 (0.43-1.80)		106 (41.1)			76 (31.4)		1.31 (1.03-1.66)^d^		1.31 (1.03-1.67)^d^				

^a^Adjusted for previous quit attempts.

^b^No risk ratio could be calculated as there were no control group participants who had no intention to quit at baseline and achieved 24 weeks of abstinence.

^c^Self-reported quitters included.

^d^*P*<.05.

### Quality of Life

Participants reported only minor health issues at baseline, with an overall score of 0.973, which slightly decreased to 0.964 at 12 months. However, there were no statistically significant differences between the intervention and control groups (*P*=.17 and *P*=.91, respectively). Furthermore, the area under the receiver operating characteristic curve remained similar between the intervention (0.971) and control (0.967) groups.

## Discussion

### Principal Findings

The supplementary intervention, comprising personalized regular messages and interactive chatting, did not lead to a significant increase in validated abstinence, self-reported 7-day PPA, smoking reduction (including quitters) by 50%, or quit attempts at both 6 and 12 months, when compared with receiving SMS text messages on general health in addition to brief SC advice and AR. While the intervention group exhibited a significantly higher 24-week continuous abstinence rate at 6 months compared with the control group, this difference was attenuated at the 12-month mark. However, more participants in the intervention group reported using SC services, with a larger effect size observed among those who expressed an intention to quit at baseline.

This study represents one of the few RCTs that provided SC support through a promising yet underutilized mobile health (mHealth) channel—mobile IM apps. Unlike SMS text messaging–based interventions that used only text and can only respond to predefined keywords, this IM-based intervention, guided by the social cognitive theory and the transtheoretical model of change, was integrated into a multicomponent, multimedia, proactive, interactive treatment model. The successful implementation of this intervention in community smokers, many of whom were not actively seeking treatments, suggests that existing SC services could consider integrating IM as an alternative channel to provide SC support. However, while the multicomponent treatment model demonstrated feasibility, the complex design of this parallel 2-group pragmatic trial limited our ability to isolate the contribution of individual components to cessation outcomes and to assess potential synergies among different components. Factorial designs, wherein participants are randomly assigned to receive either control treatment, IM-based support, AR to SC services, or a combination of these, are necessary to evaluate the additive and interactive effects of the individual components.

Compared with previous studies involving IM-based interventions [7-10,13], we have enhanced our approach by developing 3 sets of messages tailored to the planned quit date. Additionally, we have personalized the messages based on participants’ sociodemographic characteristics and smoking behaviors. In contrast to previous studies where the control group typically received minimal treatment [7,10,13], we took a proactive approach by referring all participants in this study to free and effective local SC services. This design was chosen because AR is locally recommended, such as through an online training course funded by the Department of Health for general practitioners, backed by evidence from our prior trials in community smokers [29-31]. By offering AR to both groups, it is possible that the quit rate was boosted, which might have diminished the effectiveness of the additional IM-based intervention.

Efforts to enhance the IM-based intervention included promoting the use of SC services through regular messages and chatting with SC advisors. However, these efforts faced challenges due to social unrest and the COVID-19 pandemic, leading to a delayed increase in SC service utilization. The social unrest in Hong Kong erupted in June 2019, disrupting SC services and leading to ongoing constraints in their operations until the conclusion of this RCT in October 2020. Additionally, 3 waves of COVID-19 outbreaks further compounded the situation, with related social distancing measures such as work-from-home arrangements, bans on public gatherings of more than 4 people, and the closure of many businesses [49]. The provision of SC services in Hong Kong has been further strained by limited resources, exacerbated by factors such as the limited number of centers/clinics serving all 18 districts of Hong Kong. Additionally, most of these facilities operate only during daytime working hours on weekdays, further constraining access to SC support. The prolonged disruption caused by the social unrest and the COVID-19 pandemic could have significantly impacted up to 396 participants recruited in or after May 2019. This is evident in the lower rate of SC service usage at 3 months, which reached a similar level at 6 months [30,31].

The collaboration between one of the local SC providers and the Department of Health, Hong Kong SAR, to implement a pilot fully remote SC treatment is a proactive response to the service disruptions caused by the COVID-19 pandemic. This initiative, which involves phone/videoconferencing counseling and e-mailing NRT since mid-2020, aims to address the constraints posed by limited resources and social distancing measures. More intervention group participants used SC services between the 6- and 12-month marks, a contrast to another RCT that did not use IM to enhance AR, which found minimal difference in service utilization between the 6- and 18-month periods [30]. An uptick in service usage between the 6- and 12-month marks implies that the intervention might have yielded delayed effects on the adoption of SC services. Conducting an additional follow-up at 15- or 18-month intervals could help corroborate this observation.

The post hoc analysis uncovered that engagement, particularly through chatting with SC advisors, seemed to have positively influenced cessation outcomes. This finding aligns with observations from our previous RCT, which offered chat-based support, indicating that chatting could serve as a moderately effective strategy for promoting SC. Although not directly comparable, we did not witness a significant increase in engagement, with nearly 20% engagement observed in both RCTs (74/350, 21.1%, in this study), despite implementing 3 sets of messages tailored to the planned quit date and further personalization involving multimedia. This could be attributed to the fact that many of the recruited smokers had low motivation to quit. A lower motivation to quit has been found to be associated with less desire to use mHealth interventions for quitting [13,50]. We have conducted in-depth qualitative interviews with participants, and the related results, especially from those who did not chat with SC advisors, will be presented elsewhere.

As chatting with SC advisors remained uncommon, regular IM messages appeared to provide no substantially better support than SMS text messages on general health, in addition to brief SC advice and AR. While the mechanisms underlying how messages improve quitting remained understudied, 2 qualitative studies suggested the “feeling of being cared for,” that is, the perception of social support, as a potential mechanism [51,52]. The messages also served as a reminder that the individual was trying to quit. Such perceptions of messages suggest that even if the messages are not about SC, they may still increase the quit rate to a certain extent, which appears to be consistent with our observation that control group participants rated the SMS text messages on general health as similarly helpful as did the intervention group participants who did not chat with SC advisors.

### Limitations

Our study had some limitations. First, only about two-fifths of participants were not affected by social unrest and the subsequent COVID-19 pandemic in Hong Kong. We conducted post hoc analyses on quitting outcomes in these participants (results not shown) but found no significant between-group differences given a much smaller sample size than the target. We observed some insignificant increases in quit attempts and smoking reduction rates, but there was insufficient statistical power to confirm these trends given the current sample size. Third, we were unable to cross-check the records of using certain SC services due to administrative reasons, but it is worth noting that we referred fewer than 10 participants to those services. We only had a basic record of whether a participant used their chosen service without more detailed information, such as the number of sessions attended and the medication/NRT received. This limitation prevented further in-depth analysis. Although we achieved an acceptable retention rate of over 60% at 12 months (446/700, 63.7%), we cannot rule out nonresponse bias. We conducted multiple imputation analyses, which yielded results similar to those from the intention-to-treat analyses. Only about two-fifths of the self-reported quitters underwent biochemical validations, a proportion similar to that observed in our recent RCTs of community smokers [13,31], albeit still suboptimal. Because of concerns about potential COVID-19 transmission from mask removal during the exhaled CO test, we implemented stringent infection control measures. These measures included disinfecting the piCO Smokerlyzer immediately before and after each test and maintaining a distance of at least 1.5 m in outdoor venues where the tests were conducted. Additionally, we excluded nondaily smokers from the study, despite the low prevalence of nondaily smoking in Hong Kong (0.6%).

### Conclusions

Our RCT demonstrated that an IM-based intervention, featuring personalized psychosocial support and assistance in utilizing SC services alongside brief advice and AR, did not yield additional improvements in quitting rates amidst the challenges posed by social unrest and the COVID-19 pandemic in Hong Kong.

## Appendix

Multimedia Appendix 1 Terms for the literature search.

Multimedia Appendix 2 Full results of the literature search.

Multimedia Appendix 3 Study protocol.

Multimedia Appendix 4 CONSORT (Consolidated Standards of Reporting Trials) checklist.

Multimedia Appendix 5 Messages for the intervention group.

Multimedia Appendix 6 Messages for the control group.

Multimedia Appendix 7 Sociodemographic characteristics at baseline, sensitivity and post hoc analyses results, and perceived helpfulness of messages.

## Abbreviations

AR: active referral
ARR: adjusted risk ratio
AWARD: Ask, Warn, Advise, Refer, and Do-it-again
CO: carbon monoxide
CONSORT: Consolidated Standards of Reporting Trials
mHealth: mobile health
NRT: nicotine replacement therapy
PPA: point prevalence abstinence
RCT: randomized controlled trial
RR: risk ratio
SC: smoking cessation
SNOSE: sequentially numbered, opaque, sealed envelope

## Data Availability

The data sets generated during or analyzed during this study are available from the corresponding author upon reasonable request.
