# Supplementary material for: Effect of Adding Personalized Instant Messaging Apps to a Brief Smoking Cessation Model in Community Smokers in Hong Kong: Pragmatic Randomized Clinical Trial
Source: J Med Internet Res. 2024 May 13;26:e44973. doi: 10.2196/44973 (PMC11130779; doi:10.2196/44973)
Supplement: Multimedia Appendix 1 [file jmir_v26i1e44973_app1.docx]

Appendix 1. Search for published or registered randomized controlled trials on PubMed and Cochrane library

| PubMed |
| --- |
| Keywords: ((tobacco[Title/Abstract]) OR (smoking[Title/Abstract])) AND ((quitting[Title/Abstract]) OR (cessation[Title/Abstract])) AND ((instant messaging[Title/Abstract]) OR (text messaging[Title/Abstract])) |
| Result filters: Clinical Trial, Meta-Analysis, Randomized Controlled Trial, Review, Systematic Review |
| Results: 124 publications |
|  |
| Cochrane library |
| Criteria: instant messaging in Title Abstract Keyword OR text messaging in Title Abstract Keyword - with 'Tobacco Addiction' in Cochrane Groups (Word variations have been searched) |
| Results: 8 reviews and 356 trial registries or publications |
