# Supplementary material for: Effect of Adding Personalized Instant Messaging Apps to a Brief Smoking Cessation Model in Community Smokers in Hong Kong: Pragmatic Randomized Clinical Trial
Source: J Med Internet Res. 2024 May 13;26:e44973. doi: 10.2196/44973 (PMC11130779; doi:10.2196/44973)
Supplement: Multimedia Appendix 3 [file jmir_v26i1e44973_app3.docx]

STUDY PROTOCOL

# Project Title:

Personalized support using instant messaging applications to increase smoking cessation in smokers proactively recruited from smoking hotspots in Hong Kong: a pragmatic randomized controlled trial

# Investigators

**Principal Investigator**

Dr. Man Ping WANG, Assistant Professor, School of Nursing, HKU

**Co-investigator**

Prof. Tai Hing LAM, Chair Professor, School of Public Health, HKU

Dr. Daniel Sai Yin HO, Associate Professor, School of Public Health, HKU

Dr. William Ho Cheung LI, Associate Professor, School of Nursing, HKU

Dr. Carlos King Ho WONG, Assistant Professor, Department of Family Medicine and Primary Care, HKU

Dr. Derek Yee Tak CHEUNG, Research Assistant Professor, School of Nursing, HKU

Dr. Jay Jung Jae LEE, Research Assistant Professor, School of Nursing, HKU

# Aims of the study

1. To assess the main effect of the personalized support using instant messaging applications (Intervention) vs. Control group on biochemical validated smoking abstinence at 6-month and 12-month.

2. To assess the effects on self-reported past 7-day abstinence, 24-week continuous abstinence, smoking reduction, self-efficacy of quitting, intention to quit, quit attempts and SC medications and services use at 6-month and 12-month.

3. To identify the mediators, which can inform mechanisms of the intervention on SC.

4. To evaluate the cost-effectiveness of the above interventions.

5. To understand the effects subjects’ experience of IM support on SC using a qualitative

study approach.

# Outcome measure(s):

- 1. Primary outcome is biochemically validated abstinence (exhaled carbon monoxide and saliva cotinine) at 6-month and 12-month follow-up.
  2. Secondary outcomes include: self-reported past 7-day abstinence, 24-week continuous abstinence, smoking reduction, self-efficacy of quitting, intention to quit, quit attempts and SC medications and services use at 6-month and 12-month.

# Scientific/historical background

Mobile phone SC intervention

Mobile health particularly text-messaging is now a part of World Health Organization’s (WHO) strategies on combating smoking (http://www.who.int/tobacco/mhealth/en/) and have been used in many countries given its low cost and popular use. Three important large trials [12-14] in the UK & US showed the effectiveness of text messaging-based intervention and the results were supported by recent systematic reviews [15, 16] with small to medium effect size ranging from 1.38-1.83 among smokers recruited from various sources, e.g. clinical settings, online platforms and advertisements. Detail mechanism analysis suggested that psychosocial support (e.g. increasing confidence, providing a sense of caring) are more important than providing outside resource information (e.g. information about existing treatments) [17]. This converges with qualitative findings that text-messaging helped remind quitting, reasons and benefits of quitting, and provided emotional support [18]. Smokers received personalized structured replies according to the preset keywords (e.g. “CRAVE”, “REASON”, “HELP”, etc.) they entered [16]. Such “semi-interactive messaging design” is important to increase interaction but can be further improved particularly for enhancing psychosocial support which requires more intensive two-way communication. Recent advancement of information communication technologies (ICTs) provides an opportunity to strengthen the interactive, personalized and synchronous responses.

Instant messaging application (IM Apps, WhatsApp and WeChat)

Theoretically, using the computer to provide counselling is possible with the technologies on deep learning and natural language process. There are several commercial programs on SC, e.g. Facebook Message (https://chatbottle.co/bots/stopsmoking-1) but with unclear content design and effectiveness. While it may be premature in this stage to use computer Chatbot widely for SC, one-to-one synchronous responses through IM Apps by trained counsellors or advisors is feasible and will provide unstructured conversation data for future machine learning to systematically develop the automated response system (e.g. Chatbot). Indeed, one-to-one synchronous text-based dialogue system has been applied in medication adherence, appointment reminder and mental health interventions [19, 20]. WhatsApp and WeChat are the two most popular IM Apps in Hong Kong and both allow sending interactive messages such as text, photo, video, animation and file, thus is the most preferred Apps for IM SC intervention in the present proposal.

Proactive recruitment of smokers in the community

Previous text-messaging-based trials recruited smokers from internet as these smokers were more likely to use the application. We propose to recruit smokers from the community for several reasons. IM Apps are used widely in HK. It is very unlikely community smokers have no IM application (please refer to feasibility for details). Smokers in the community constitute the majority of smokers and are those most in need for SC interventions as most of them have never tried any SC service. This also builds on our developed systematic methods to recruit smokers at smoking hotspots (SH), which are defined as public outdoor places where smokers stop/linger and smoke [5, 9]. In the past 6 years, we have consecutively recruited more than 6000 smokers in the community. Briefly trained (1-day workshop) health care student helpers (nursing, medical, pharmacy) approached and recruited smokers in SH. We adopted “a-foot-into-the-door” approach by asking smokers a few simple questions related to smoking. Those who answered the questions were further invited to participate in the SC trials. About 10-20 smokers can be recruited in each session (about 6 hours). Although the subjects recruited may not be representative of the general smoking population, we found small differences (Cohen’s effect size of difference between community recruited smokers and smokers from the general population for age, sex, cigarette consumption and quit attempt <0.2) [21]. This method is particularly efficient in places where smokefree legislation bans smoking in most indoor places (e.g. HK and many major cities in the world) and smokers have to smoke in public open places.

Brief SC advice (AWARD)

Behavioral or psychological counselling is effective for SC but mostly consist of multi-session face-to-face or telephone counselling [22, 23]. Brief intervention (e.g. 5As: Ask; Advise to quit; Assess willingness to make quit attempts; Assist in quit attempts, Arrange follow-up) delivered by physicians or nurses is suggested in the US & UK SC guidelines. A systematic review found brief advice alone was effective in increasing quit attempts [3] which is an important precursor of successful quitting. The 5As or 5Rs (Relevance, Risks, Rewards, Roadblocks, Repetition) interventions usually take 5-10 minutes to complete. The UK National Centre for Smoking Cessation and Training has developed a shorter intervention of very brief advice (VBA) which requires only 30 seconds to conduct 3As: Ask, Advise and Assist [11]. There is no RCT on the effectiveness of VBA (or 3As) on smoking abstinence. Based on the 5As/5Rs and other validated SC intervention models, and before the 3As, we have developed and validated a brief intervention, the AWARD model (Ask, Warn, Advise, Refer, Do-it-again) (<5 minute) delivered by trained non-health care professionals (e.g. nursing students) to smokers in the community [4-10]. Thus we propose to enhance our established AWARD model with a quantum leap, by incorporating IM Apps at follow-up interventions (e-AWARD).

Feasibility supported by a qualitative study and pilot trial

HK is one of the cities with the highest smartphone coverage and data connection [24]. IM Apps usage is very popular and majority use WhatsApp or WeChat. We have conducted 5 focus group interviews on 22 smokers (68% male, 55% daily smokers, mean age 49) to explore their acceptability and expected intensity and frequency of instant messages for SC (HKU/HA HKW IRB no. UW 17-206). After transcription and coding, we have found using IM Apps is highly regarded as a suitable, useful and personalized intervention to encourage smokers to quit smoking. The findings are being written up for publication with some quotes, which are shown below: “WhatsApp is better than telephone, you can choose when to reply and not being disturbed by telephone calls” [C4, male, 38yrs], “WhatsApp messages can remind you, encourage you when you need is particularly suitable for people like me” [B4, male, 42yrs], “I think you can discuss with the smokers in a personalized context for example according to the smokers’ work environment … and family background is very important” [B5, male, 25yrs]. The findings also inform the IM Apps design in this proposal (please ***6.6***). Smokers also expect that the frequency and duration of regular messages can be tailored to their needs (e.g. quit date) and they can choose the frequencies and duration.

We have conducted a trial under the context of “Quit-to-Win” to test the feasibility of using WhatsApp IM on supporting SC among community smokers (Clinical Trial ID: NCT03182790). Among smokers we approached, about 95% used IM Apps daily. At 1-month follow, among 156 smokers randomized to receive IM (AWARD baseline plus IM psychosocial support), 96% received and read the messages (only 3 did not read because they were not interested or too busy), 31% chatted with the counsellors at least weekly. Many smokers perceived the messages helped increase motivation to quit (66%) and quit attempt (57%). Smokers generally were satisfied with the interaction with the counsellor (score 8.3/10). Preliminary results showed higher self-reported quit rate in intervention (9.0%) compared with control (5.3%) (AWARD advice only) at 1-month follow-up.

# Study design

## 6.1 Subjects

**Inclusion criteria:**

1) Adult smokers aged 18+ who smoke cigarette(s) daily.

2) Exhaled carbon monoxide (CO) level of 4ppm or above.

3) Having smartphones with IM Apps (WhatsApp or WeChat) and have experience in using.

4) Hong Kong residents able to read and communicate in Chinese (Cantonese or Putonghua).

**Exclusion criteria:**

1) Smokers who have psychiatric/psychological diseases or are on regular psychotropic medications.

2) Smokers who are using SC medication, NRT, other SC services or projects.

## 6.2 Settings

A smoking hotspot (SH) is defined as a public outdoor place where smokers stop/linger and smoke [5, 9]. We aim to include a reasonably “representative” or unbiased sample of SH at different locations from all three regions of Hong Kong. A sampling frame of all SH is not available and cannot be established within this proposal. We have identified 15 hotspots in a previous community trial and successfully recruited more than 750 smokers in 4 months in 2015. Based on our previous experience, the locations of SH include places near exits of underground and railway stations, shopping malls and large commercial buildings [5]. To include more and new SH for intervention, we will select 15 more SHs from the field observation results of public open places across the three major regions (Hong Kong Island, Kowloon and New Territories) making up a total of 30 SH for recruitment. Two trained observers will count pedestrian flow, the number of smokers and assess the eligibility, which includes the appropriateness of the environment for delivering intervention (e.g. smokers’ duration of stay, noise level, space) using standardized forms, to be improved from the form we have designed and used before.

***6.3 Subject Recruitment***

Two trained SC advisors (student helpers) and one supervisor (experienced research assistant) will be deployed for each session of intervention at an SH. Potential subjects (Chinese adult smokers who linger/stop at the SH) will be approached using the “a-foot-into-the-door” approach in which SC advisors will ask if the smoker would like to reduce/quit smoking and receive SC intervention or advice. If there is more than 1 potential subject, SC advisors will randomly select 1 smoker to avoid contamination (as they may share information from IM Apps subsequently). Smokers will be assessed for eligibility and informed written consent will be sought. Subjects will complete a brief self-administered questionnaire (baseline). To increase IM App intervention compliance and reduce later hang-ups of telephone surveys, SC ambassador will save the contact phone number of the trial into subjects’ mobile phones. This has been found to be feasible in our pilot trial which reached 80% at 1-month in 2017.

***6.4 Randomization and allocation concealment***

Sequentially numbered, opaque, sealed envelopes (SNOSE) and block randomization will be used to individually allocate subjects into 2 groups with equal size. The co-investigator (DYTC, statistician) will prepare 696 identical, opaque, sealed, A5-sized envelopes. Half of them (n=348) will each contain the cards for the intervention and controlled group. Blocks of 4, 8, and 12 in random order will be created with equal numbers to be allocated to the 2 groups. All envelops will be labelled with serial numbers and the SC ambassadors will be totally concealed about the random allocation sequence. Once a smoker has signed the consent form, an SC advisor will open one SNOSE according to the serial number to determine the group allocation. To avoid intervention contamination, each intervention will be delivered to one smoker at a time. Upon the completion of the first intervention, there will be a 5-minute interval to let the recruited smoker leave, before the next recruitment of a new smoker at the same SH. This approach will minimise the chance of the recruiting smokers who have a connection with the previous subjects.

***6.5 Interventions for intervention group***

Subjects in the intervention group will receive brief SC advice using ***AWARD*** model. After ***A***sk, face-to-face oral, ***W***arning, ***A***dvice and ***R***eferral (***WAR)*** will be conducted by the SC advisors within about 2 to 5 minutes, a leaflet (A5 size) (Appendix 5) will be given to the smoker. Smokers will receive IM Apps regular message and psychosocial support for 12-week. Details of AWARD and IM Apps intervention are as below. ***AWARD model***

1. ***Ask:*** smoking habit, quit intention and attempt, smoking reduction intention, previous quitting experience including NRT and SC use.

2. ***Warn***: smokers will be orally warned about the harms of smoking and receive a leaflet, which includes some shocking pictures of diseases due to smoking, and SC information (Appendix 5). WHO’s warning that “1 of 2 smokers will be killed by smoking,” and the risk could be 2 out 3 in smokers who started smoking at young age and smoke heavily will be emphasized in oral warning and the leaflet.

3. ***Advise***: smokers will be advised to quit as soon as possible and use NRT or SC services.

4. ***Refer***: smokers will be advised to buy their own NRT or seek SC services for free NRT. Subjects who prefer to receive further counselling or treatment and free NRT will be actively referred to the Tung Wah Hospital Groups of Integrative Centre Smoking Cessation (ICSC), which is the largest free SC service provider in Hong Kong, Po Oi Hospital for traditional Chinese acupuncture, Department of Health Smoking cessation quitline, Women quitline (HKU), Youth quitline (HKU) (for those aged 18-25) or Hospital Authority smoking cessation clinics. Active referral means that the collected contact information with consent will be sent to the preferred SC services providers for a quick appointment and follow-up. We will liaise with the all SC providers for progress monitoring and data collection on service use. Our published findings showed active referral group had an odds ratio of 1.81 (95% CI 1.04-3.16) for biochemically validated quitting at 6-month compared with the control group [10].

5. ***Do-it-again***: smokers who have tried to quit but relapsed will receive the ***WAR*** (Warn, Advise, Refer) intervention and be encouraged to try to quit again or reduce smoking. Smokers who have reduced cigarette consumptions (but have not quit) will be advised to further reduce or quit smoking (cut-down-to-quit), which is based on evidence from our previous trial [6]. Smokers who wish to have further in-depth counselling will be referred to experienced SC providers. This will be carried at the follow-up interventions at 3-month before the telephone survey for the Control group. or the Intervention group, this will be delivered using the IM Apps (please see ***6.6***) and at 3-month before the telephone survey.

***6.6 IM Apps interventions***

**Theoretical frameworks**

Social Cognitive Theory (SCT) and Transtheoratical Model (TTM) have been used in the previous text messaging-based interventions for SC [13, 14]. We propose to use these theoretical frameworks to guide the design of regular messages and synchronous IM Apps psychosocial support. SCT posits that personal factors (cognitive, affective and biological events) and environmental factors affect behavioural changes [25]. The regular messages and IM psychosocial support will aim to increase self-efficacy, social support and behavioural capacity of quitting, and to clarify the outcome expectation of quitting. Practical skills and emotional support on managing cravings, withdraw symptoms, situational triggers, lapse and relapse will be designed guided by SCT with reference to standard information from HK Department of Health smoking cessation kit [26] and our evidence from a smoking relapse prevention trial [27]. TTM [28] is popularly used in SC studies and we are experienced in using TTM in many of our SC trials [29-31]. By using TTM, messages and psychosocial support will be designed according to smokers’ stages of change e.g. pre-contemplation, contemplation, preparation, action, and maintenance or relapse.

**Regular messages through IM Apps (WhatsApp or WeChat)**

Personalized regular messages will be designed based on the recommended steps by Abroms [32], our qualitative study and feasibility trial. Personalization is the key to lead central process of the information by smokers as suggested by the Elaboration Likelihood Model [33]. Tailor messages will be based on sociodemographic characteristics (surname, gender, occupation), smoking habit at baseline (tobacco dependence level, motivation to quit and self-efficacy) and updated smoking status obtained during IM Apps conversation (smoking, abstinence or relapse). Among smokers who can set a quit date at baseline or during subsequent follow-up conversation through IM Apps, messages on reminding the quit date will be sent before the quit date and motivational messages to encourage abstinence in subsequent days (please see below).

A total of pre-set 26 messages will be sent with the schedule of once daily for 1 week (the week across the quit date), 3 times a week for 4 weeks (2 weeks each before and after the week with quit date) and once a week for remaining 7 weeks. The schedule will be adjusted according to the quit date (if quit date not selected, the daily messages will start at the beginning of follow-up) and also smokers’ requests during IM Apps conversation to increase or reduce the adjust frequency or timing of receiving the messages. The format of regular messages will mainly be text-based but also include pictures, animations and videos to attract reading as found in our qualitative study. The proposed total number of regular messages is less than previous text messaging-based trials [12-14] as synchronous psychosocial support will also be provided in present proposed project.

**Synchronous psychosocial support through IM Apps (WhatsApp/WeChat)**

The aim of synchronous psychosocial support is to provide personalized interactive IM Apps text/voice conversation by trained SC advisors to motivate smokers to initiate quitting and walk through the quitting process with smokers. This is an extension of our baseline face-to-face AWARD intervention for 3 months. One of the most important functions of IM Apps is to allow advisors to provide timely responses to smokers’ messages. For example, to provide support to avoid or handle high risk situations of smoking (e.g. cigarette invitation from friends, stressful events, boredom); to break the habitual smoking by timely appropriate event-oriented messages (e.g. first cigarette in morning, smoking after meals, smoking during breaks at work). Other than relying on regular messages, advisors will also periodically proactively send IM messages to initiate the conversation (e.g. asking recent progress of SC) and deliver evidence-based advice guided by the SCT and TTM. Advisors will actively refer smokers, if they have expressed the need, to SC services providers. A standard operation algorithm for SC advisor used in our pilot feasibility trial will be modified for the use.

We plan to train (please see ***6.13***) 15-20 SC advisors (1 for about 10 smokers for 3 months). There will be no restriction on the intensity of IM Apps conversation between smokers and advisors will only be available during day time and during working days to prevent overburden. According to our pilot feasibility trial among 156 smokers, most interacted with advisors for 2-3 conversations a day, lasting for about 1-2 weeks with several messages in subsequent weeks. Each advisor will be supervised by our senior SC counsellors in our team who will provide immediate support and help in handling difficult cases if any. All conversation content will be recorded for data analysis and quality monitoring (below).

***6.7 Intervention for Control Groups***

The Control group will receive the same interventions as Intervention group at baseline. At follow-up, they will receive regular message (using SMS) with similar frequency to Intervention group but with content on **general health** and reminding the importance of participating in the follow-up surveys and biochemical validation for quitting. Our previous trial found regular SMS message on general health did not affect quitting [4].

|  | Intervention | Control |
| --- | --- | --- |
| **Baseline intervention** |  |  |
| AWARD advice, health warning leaflet, active referral | ✓ | ✓ |
| **Follow-up intervention** |  |  |
| IM regular messages, psychosocial support & referral | ✓ |  |
| SMS message on general health & follow-up survey reminders |  | ✓ |

***6.8 Summary of interventions for intervention group and control group:***

***6.9 Outcomes***

The primary outcomes are CO validated (<4 ppm) smoking abstinence at 6-month and 12-month follow-up which are the gold standard to determine abstinence in many SC trials [23, 34]. Secondary outcomes include self-reported 7-day point prevalence and continuous abstinence for 24-week, quit intention and attempts, smoking reduction, nicotine addiction level (Heaviness Smoking Index), NRT use, SC service use at 6-month and 12-month. Smoking reduction is defined as self- reported reduction in the number of cigarettes smoked daily by at least 50% of the baseline amount, as in our previous publications [1]. This will be calculated by including and excluding quitters. At baseline and 12-month, validated Chinese five-level EuroQol five-dimensional questionnaire (EQ-5D-5L) [35] will be used to calculate EQ-5D-5L health utility scores. Using the health utility scores at baseline and follow-up, quality-adjusted life year (QALY) will be estimated by our health economist (Dr Wong CK) for incremental cost-effectiveness ratios of the intervention when compared to control (please see ***6.12***). Outcome assessors conducting the follow-up surveys will be blinded to the group allocation.

***6.10 Measurements***

|  | Baseline | 3M | 6M | 12M |
| --- | --- | --- | --- | --- |
| Socio-demographic characteristics& smoking^1^ | ✓ |  |  |  |
| Smoking, quitting and reduction behaviors^2^ | ✓ | ✓ | ✓ | ✓ |
| Self-efficacy, cessation service, NRT use^3^ | ✓ | ✓ | ✓ | ✓ |
| CO validation of quit |  |  | ✓ | ✓ |
| Quality of life (EQ-5D-5L) | ✓ |  |  | ✓ |

Data (as in the following table) will be collected at 3, 6 and 12 months after randomization using telephone surveys and face-to-face CO validation. Incentives of HK$ 50 coupons will be sent by mail (or using electronic coupons) to those who have completed each survey at 3, 6 & 12 months (total $150). HK$ 300 will be provided for those have completed each CO validation to compensate for travel expenses and time (total $600). Our experiences show that such an amount can increase response rate substantially. We will use the tablet PCs installed with Computer Assisted Patient Interview (CAPI) system to assist in collecting data at SH and also at the follow-up surveys. The CAPI has been used in our Mega-RCT project (NCT02494960) and has collected data from more than 13736 smokers in Guangdong, China. The CAPI has built-in functions against errors and data collected can be easily generated for progress monitoring and analysis. This will largely reduce the costs of data entry and cleaning, and is environmentally friendly. An electronic questionnaire will be designed using the brief (< 2minutes) and validated questions (which have been used in many of our previous RCT papers and by others) [36-39] to measure the outcomes, and to maintain a higher retention rate and applicability in real-world practice. Our research team has extensive experience in conducting the exhaled CO validation tests (Smokerlyzer). The questionnaire content is summarized as follows:

^1^ Age, education level, year of smoking.^2^ Number of cigarette consumed daily and time of the first cigarette after weak-up in the morning (to calculate Heaviness of Smoking Index), quit intention (yes/no), the number of quit attempts in past 30 days. Past 7-day smoking abstinence and continuous abstinence for 24-week. Smoking reduction intention and attempts.^3^ Recent use (past 24-week) of cessation medication, counselling, hotline, clinics, other smoking cessation programs.

***6.11 Sample size calculation***

As there is no trial in the literature about IM Apps and SC, we have calculated the sample size based on similar trials and our previous trials using AWARD intervention. Based on our previous trials, the biochemically validated quit rate for the Control group was about 5% at 6-month follow-up [4, 10]. Based on the previous trial on text messaging-based interventions, the 6-month biochemically validated quit rate was 10.7% [12]. Both studies used the intention-to-treat principle for calculating the quit rates. Using this quit rate of text messaging-based intervention for IM intervention may underestimate the effect and is conservative. With the type I error 0.05, power 80% and allocation ratio 1:1, the required sample size for determining a significant group difference of 6-month biochemically validated quit rates between IM Apps Intervention group and Control group is 696 (each group: 348 subjects).

***6.12 Statistical and qualitative analysis***

Baseline characteristics between 2 groups will be compared to check for variables with large differences, which, if found, need to be adjusted in comparing outcomes between groups. Intention-to-treat (ITT: the standard and most conservative approach in RCT) analysis will be used by imputing all non-responses at follow-up by baseline values, i.e. assuming failures or no changes after intervention, to yield more conservative estimates of effective size.

The **primary analyses** include: (1) Main effect: Intervention vs. Control on biochemically validated abstinence at 6-month and 12-month

The **secondary analyses** include: (2) Main effect adjusting for baseline difference; (3) All secondary outcomes (see ***4***) at 6-months and 12-months; (4) Mediation analysis of 3-month factors (psychosocial or resource effects) on biochemically validated abstinence at 6-month (and 3-month & 6 month factors on 12-month outcomes); (5) Subgroup analysis based on intention to quit at baseline; (6) Cost-effectiveness analysis over the 12-month trial period and long-term cost-effectiveness analysis over the lifetime horizon (please see below); (7) Qualitative study data analysis for understanding the effects of the intervention. Sensitivity analyses (e.g. complete case or per protocol) will be conducted, depending on the actual pattern of missing data, with different methods of statistical imputation (e.g. multiple/simple imputations/Last Observation Carried Forward) to assess the robustness of the findings. For the outcomes from multiple time points (3-, 6- and 12-month), generalized linear mixed models which allow for multiple observations between subjects and within subjects, will be used. Main effect and interaction effect will be included.

Cost-effectiveness analysis (CEA) of the intervention will be conducted using standard methods [40] by health economist (Co-I Dr Wong CK). For short-term cost-effectiveness, a decision tree will be developed using the empirical RCT data to evaluate the effect in 12-month period. An ingredient approach will be used to estimate the cost of the intervention program including intervention materials (e.g. leaflet), administration fee, and time for SC ambassadors to deliver the intervention, while the healthcare resource use with respect to general and specialist outpatient visits, length of hospital stay, emergency visits will be measured for each subject in the Intervention and Control groups. The health effectiveness outcomes will include the number of quitters at 6-month and quality adjusted life year (QALY) gained. EQ-5D-5L utility scores at baseline and follow-up assessments will be used to construct QALY using area under the receiver operating characteristic curve approach. For long-term cost-effectiveness simulation, a Markov model will be developed using the 12-month quit rate estimate taken from the RCT to model the lifetime effects of the intervention on total costs and total QALY [41]. A perspective of healthcare provider will be taken and 3% discount rate will be applied. Treatment costs for smoking-related morbidities (e.g. COPD, CHD, lung cancer, stroke, asthma) and relative risks will be extracted from the literature and costs of the intervention derived from the RCT. Incremental cost-effectiveness ratios (ICERs) in the form of incremental cost per incremental QALY gained from intervention will be calculated over the time frames of 5, 10, and 20 years and the lifetime of the simulated cohort. One-way and probabilistic sensitivity analyses will be conducted to test the robustness of the model.

A qualitative approach will be adopted for understanding experience of subjects in the intervention group (by qualitative researcher Co-I Dr. Lee JJ). The subjects will be recruited from subgroups based on smoking status at 12-month (quit, not quit) and IM Apps conversation involvement (>10 or <10 times). At least 20 (N=5 for each subgroup) participants will be interviewed and final sample depends on data saturation. Individual semi-structured interviews will be conducted. All interviews will be audio-recorded and transcribed verbatim. The data analysis process will begin immediately after each individual interview in accordance with a thematic analysis framework introduced by Braun & Clarke [42] using NVivo 11. Codes, categories and themes generated will be compared with the established taxonomy for evaluating intervention quality related to behavioural change technique (CBT) for smoking cessation [43].

***6.13 Training of SC advisors & intervention fidelity***

About 15-20 undergraduates from health care disciplines will be recruited to participate in the project as SC advisors. They will be trained in one-day workshops on various aspects of knowledge and skills related to SC, and specific skills on IM Apps use and responses by experienced IM researchers. We have extensive experience on short and intensive SC training in many of our previous SC projects using standard principles from AHCPR & WHO [44, 45]. Those who pass the test (written) will be qualified as an SC advisor. All the procedures will be clearly instructed using standard operation protocols. Project supervisors (experienced SC team staff) will randomly select 10% of the recruitment sessions in the early phase of the trial for intervention fidelity checks using standardized forms. During fieldwork, our experienced SC counselor will standby as a back up to handle any questions and problems by telephone or IM Apps and conduct random fidelity checks (10%). All IM Apps conversation will be recorded and 5% will be randomly checked (with no prior notification) by experienced SC counselors for quality control.

# Estimated duration and commencement date

Proposed starting date: 1 April 2018

Proposed study completion date: 31 March 2020

Expected final report date: 30 June 2020

# Direct access to source data/documents

The raw data will be stored on an external hard-disk and locked in a cupboard with keys kept by the Principal Investigator. Only the Investigators and Research Assistant of the project will be permitted to access the raw data and/or study records. The data will be scanned and kept for 10 years or longer after the study is completed. Individual participants will not be directly identifiable from the dataset to be used for analysis.

# Consent

Participation in the study is totally voluntary. The SC advisors at the study sites will explain to smokers that we are carrying out a study on smoking cessation. The SC advisors will explain to the participants that they will receive telephone calls at 3, 6 and 12 months for the follow-up of their smoking status. The participants will be assured that they can withdraw from the study anytime without any prejudice, and all the information will be kept confidential and results will be reported in an aggregate format. Agreement to participate in the RCT will be considered as consent and participants are required to sign the written consent form.

# Conflict of interest: None

# References

1. Lam TH, Cheung YT, Leung DY, Abdullah AS, Chan SS. Effectiveness of smoking reduction intervention for hardcore smokers. Tob Induc Dis. 2015;13(1):9.

2. Census and Statistics Department. Thematic Household Survey, Report No.59: Pattern of Smoking Hong Kong SAR: Hong Kong SAR Government. 2016. Available from: http://www.statistics.gov.hk/pub/B11302592016XXXXB0100.pdf.

3. Aveyard P, Begh R, Parsons A, West R. Brief opportunistic smoking cessation interventions: a systematic review and meta-analysis to compare advice to quit and offer of assistance. Addiction. 2012;107(6):1066-73.

4. Chan SS, Wong DC, Cheung YT, Leung DY, Lau L, Lai V, Lam TH. A block randomized controlled trial of a brief smoking cessation counselling and advice through short message service on participants who joined the Quit to Win Contest in Hong Kong. Health Educ Res. 2015;30(4):609-21.

5. Chan SS, Cheung YT, Wan Z, Wang MP, Lam TH. Proactive and Brief Smoking Cessation Intervention for Smokers at Outdoor Smoking "Hotspots" in Hong Kong. Journal of cancer education : the official journal of the American Association for Cancer Education. 2016; EPub, doi: 10.1007/s13187-016-1085-3.

6. Wang MP, Li WCH, Cheung DY, Wu Y, Lam OB, Kwong AC, Lai VW, Chan SS, Lam TH. Brief advices on smoking reduction vs. abrupt quit for smoking cessation in Chinese smokers: a cluster randomized controlled trial. Nicotine Tob Res. 2017; EPub, doi: 10.1093/ntr/ntx026.

7. Cheung YT, Wang MP, Li HC, Kwong A, Lai V, Chan SS, Lam TH. Effectiveness of a small cash incentive on abstinence and use of cessation aids for adult smokers: A randomized controlled trial. Addict Behav. 2017;66:17-25.

8. Chan SSC, Cheung YTD, Wong YMB, Kwong A, Lai V, Lam TH. A brief smoking cessation advice by youth counselors for the smokers in the Hong Kong Quit to Win Contest 2010: a cluster randomized controlled trial. Prev Sci. 2017; EPub, doi: 10.1007/s11121-017-0823-z.

9. Cheung YTD, Lam TH, Li WHC, Wang MP, Chan SSC. Feasibility, Efficacy and Cost Analysis of Promoting Smoking Cessation at Outdoor Smoking "hotspots": a Pre-post Study. Nicotine Tob Res. 2017; EPub, doi: 10.1093/ntr/ntx147.

10. Wang MP, Suen YN, Li HC, Lam CO, Wu SY, Kwong AC, Lai VW, Chan SS, Lam TH. Intervention with breif cessation advice plus active referral for proactively recruited community smokers: a pragmatic clinical randomized clinical trial. JAMA Intern Med. 2017; EPub, doi: 10.1001/jamainternmed.2017.5793.

11. Wright J. Very brief advice can be effective in encouraging smokers to quit 2013 Available from: http://www.guidelinesinpractice.co.uk/jan_13_wright_smoking_jan13#.Vf927RGqqko.

12. Free C, Knight R, Robertson S, Whittaker R, Edwards P, Zhou W, Rodgers A, Cairns J, Kenward MG, Roberts I. Smoking cessation support delivered via mobile phone text messaging (txt2stop): a single-blind, randomised trial. Lancet. 2011;378(9785):49-55.

13. Abroms LC, Boal AL, Simmens SJ, Mendel JA, Windsor RA. A randomized trial of Text2Quit: a text messaging program for smoking cessation. Am J Prev Med. 2014;47(3):242-50.

14. Naughton F, Cooper S, Foster K, Emery J, Leonardi-Bee J, Sutton S, Jones M, Ussher M, Whitemore R, Leighton M, Montgomery A, Parrott S, Coleman T. Large multi-centre pilot randomized controlled trial testing a low-cost, tailored, self-help smoking cessation text message intervention for pregnant smokers (MiQuit). Addiction. 2017;112(7):1238-49.

15. Whittaker R, McRobbie H, Bullen C, Rodgers A, Gu Y. Mobile phone-based interventions for smoking cessation. Cochrane Database Syst Rev. 2016;4:Cd006611.

16. Scott-Sheldon LA, Lantini R, Jennings EG, Thind H, Rosen RK, Salmoirago-Blotcher E, Bock BC. Text Messaging-Based Interventions for Smoking Cessation: A Systematic Review and Meta-Analysis. JMIR mHealth and uHealth. 2016;4(2):e49.

17. Hoeppner BB, Hoeppner SS, Abroms LC. How do text-messaging smoking cessation interventions confer benefit? A multiple mediation analysis of Text2Quit. Addiction. 2017;112(4):673-82.

18. Douglas N, Free C. 'Someone batting in my corner': experiences of smoking-cessation support via text message. Br J Gen Pract. 2013;63(616):e768-76.

19. Hoermann S, McCabe KL, Milne DN, Calvo RA. Application of Synchronous Text-Based Dialogue Systems in Mental Health Interventions: Systematic Review. J Med Internet Res. 2017;19(8):e267.

20. Hamine S, Gerth-Guyette E, Faulx D, Green BB, Ginsburg AS. Impact of mHealth chronic disease management on treatment adherence and patient outcomes: a systematic review. J Med Internet Res. 2015;17(2):e52.

21. Wang MP, Li WH, Jiang N, Chu LY, Kwong A, Lai V, Lam TH. E-Cigarette Awareness, Perceptions and Use among Community-Recruited Smokers in Hong Kong. PloS One. 2015;10(10):e0141683.

22. Lindoson-Hawley N, Thompson TP, Begh R. Motivational interviewing for smoking cessation. Cochrane Database Syst Rev. 2014;CD006936.

23. Stead LF, Hartmann-boyce J, Perera R, Lancaster T. Telephone counselling for smoking cessation. Cochrane Database Syst Rev. 2013;CD002850.

24. Thematic household survey Report No.62: Information technology usage and penetration. Hong Kong SAR Government. 2016. Available from: http://www.digital21.gov.hk/eng/statistics/download/informationsociety2013.pdf. 25. Bandura A. Social Foundation of Thought and Action: A Social Cognitive Theory. Englewood Cliffs, NJ: Prentice-Hall; 1986.

26. Department of Health. Smoking Cessation Information Kit. Hong Kong SAR: 2015. Avialabe from: https://www.tco.gov.hk/english/quitting/files/kit09_eng.pdf

27. Cheung YT, Chan CH, Lai CK, Chan WF, Wang MP, Li HC, Chan SS, Lam TH. Using WhatsApp and Facebook online social groups for smoking relapse prevention for recent quitters: A pilot pragmatic cluster randomized controlled trial. J Med Internet Res. 2015;17(10):e238.

28. Prochaska JO, Velicer WF. The transtheoretical model of health behavior change. Am J Health Promot. 1997;12:38-48.

29. Chan SS, Leung DY, Wong DC, Lau CP, Wong VT, Lam TH. A randomized controlled trial of stage-matched intervention for smoking cessation in cardiac out-patients. Addiction. 2012;107(4):829-37.

30. Li WH, Chan SS, Wang KM, Lam TH. Helping cancer patients quit smoking by increasing their risk perception: a study protocol of a cluster randomized controlled trial. BMC Cancer. 2015;15:490.

31. Li WH, Wang MP, Lam TH, Cheung YT, Cheung DY, Suen YN, Ho KY, Tan KC, Chan SS. Brief intervention to promote smoking cessation and improve glycemic control in smokers with type 2 diabetes: a randomized controlled trial. Sci Rep. 2017;7:45902.

32. Abroms LC, Whittaker R, Free C, Mendel Van Alstyne J, Schindler-Ruwisch JM. Developing and pretesting a text messaging program for health behavior change: recommended steps. JMIR mHealth and uHealth. 2015;3(4):e107.

33. Petty RE, Cacioppo JT. The elaboration likelihood model of persuasion. Adv Exp Soc Psychol. 1986;19:124-92.

34. Stead LF, Perera R, Bullen C, Mant D, Lancaster T. Nicotine replacement therapy for smoking cessation. Cochrane Database Syst Rev. 2008;CD000146.

35. Luo N, Liu G, Li M, Guan H, Jin X, Rand-Hendriksen K. Estimating an EQ-5D-5L Value Set for China. Value Health. 2017;20(4):662-9.

36. Lam TH, Abdullah AS, Chan SS, Hedley AJ. Adherence to nicotine replacement therapy versus quitting smoking among Chinese smokers: a preliminary investigation. Psychopharmacology. 2005;177:400-8.

37. Chan SS, Leung DY, Abdullah AS, Wong VT, Hedley AJ, Lam TH. A randomized controlled trial of a smoking reduction plus nicotine replacement therapy intervention for smokers not willing to quit smoking. Addiction. 2011;106(6):1155-63.

38. Chan SS, Wong DC, Cheung YT, Leung DY, Lau L, Lai V, Lam TH. A block randomized controlled trial of a brief smoking cessation counselling and advice through short message service on participants who joined the Quit to Win Contest in Hong Kong. Health Edu Res. 2015;30(4):609-21.

39. Lin PR, Zhao ZW, Cheng KK, Lam TH. The effect of physician's 30 s smoking cessation intervention for male medical outpatients: a pilot randomized controlled trial. J Public Health. 2013;35(3):375-83.

40. Cromwell J, Bartosch WJ, Fiore MC, Hasselblad V, Baker T. Cost-effectiveness of the clinical practice recommendations in the AHCPR guideline for smoking cessation. JAMA. 1997;278:1759-66.

41. Yudkin P, Hey K, Roberts S, Welch S, Murphy M, Walton R. Abstinence from smoking eight years after participation in randomised controlled trial of nicotine patch. BMJ. 2003;327(7405):28-9.

42. Braun V, Clarke V. Using thematic analysis in psychology. Qual Res Psychol. 2006;3(2):77-101.

43. Michie S, Hyder N, Walia A, West R. Development of a taxonomy of behaviour change techniques used in individual behavioural support for smoking cessation. Addict Behav. 2011;36(4):315-9.

44. The Tobacco Use and Dependence Clinical Practice Guideline Panel, Staff, and Consortium Representatives. A clinical practice guideline for treating tobacco use and dependence: A US Public Health Service report. JAMA. 2000;283(24):3244-54.

45. Sabaté E. Adherence to long-term therapies: evidence for action. World Health Organization. 2003. Available from: http://apps.who.int/iris/bitstream/10665/42682/1/9241545992.pdf
