# Supplementary material for: Effect of Adding Personalized Instant Messaging Apps to a Brief Smoking Cessation Model in Community Smokers in Hong Kong: Pragmatic Randomized Clinical Trial
Source: J Med Internet Res. 2024 May 13;26:e44973. doi: 10.2196/44973 (PMC11130779; doi:10.2196/44973)
Supplement: Multimedia Appendix 7 [file jmir_v26i1e44973_app7.docx]

Table S1. Socio-demographic and smoking-related characteristics at baseline by whether chatting with smoking cessation advisors (N=350)

|  | Chatted  N=74 | Not chatted  N=276 | P value |
| --- | --- | --- | --- |
|  | n (%) | n (%) |  |
| Sex |  |  | 0.23 |
| Male | 48 (64.9) | 199 (72.1) |  |
| Female | 26 (35.1) | 77 (27.9) |  |
| Age (years) |  |  | 0.62 |
| 18-29 | 5 (7.5) | 35 (13.7) |  |
| 30-39 | 11 (16.4) | 41 (16.1) |  |
| 40-49 | 18 (26.9) | 62 (24.3) |  |
| 50-59 | 16 (23.9) | 66 (25.9) |  |
| 60 or above | 17 (25.4) | 51 (20.0) |  |
| Highest educational attainment |  |  | 0.71 |
| Primary of below | 10 (16.1) | 31 (12.8) |  |
| Secondary | 41 (66.1) | 173 (71.2) |  |
| Tertiary | 11 (17.7) | 39 (16.0) |  |
| Marital |  |  | 0.09 |
| Single | 11 (16.4) | 74 (29.8) |  |
| Married/co-habited | 53 (79.1) | 163 (65.7) |  |
| Divorced/widow | 3 (4.5) | 11 (4.4) |  |
| Children living together |  |  | **0.050** |
| No | 35 (58.3) | 169 (71.3) |  |
| Yes | 25 (41.7) | 68 (28.7) |  |
| Housing |  |  | 0.50 |
| Rent | 35 (55.6) | 133 (53.8) |  |
| Owned | 26 (41.3) | 111 (44.9) |  |
| Others | 2 (3.2) | 3 (1.2) |  |
| Employment status ^a^ |  |  | 0.38 |
| Economically active | 43 (62.3) | 170 (68.0) |  |
| Economically inactive | 26 (37.7) | 80 (32.0) |  |
| Monthly household income (US$ 1=HK$ 7.8) |  |  | 0.36 |
| Unstable/<19999 | 31 (50.0) | 94 (40.3) |  |
| 20000-29999 | 15 (24.2) | 73 (31.3) |  |
| 30000 or above | 16 (25.8) | 66 (28.3) |  |
| Time to first cigarette after waking |  |  | 0.87 |
| After 60 minutes | 9 (13.0) | 42 (16.1) |  |
| 31-60 minutes | 9 (13.0) | 33 (12.6) |  |
| 6-30 minutes | 22 (31.9) | 72 (27.6) |  |
| Within 5 minutes | 29 (42.0) | 114 (43.7) |  |
| Daily cigarette consumption |  |  | 0.73 |
| 1-10 | 38 (51.4) | 123 (44.7) |  |
| 11-20 | 28 (37.8) | 119 (43.3) |  |
| 21-30 | 4 (5.4) | 20 (7.3) |  |
| 31 or above | 4 (5.4) | 13 (4.7) |  |
| Previous quit attempt |  |  | 0.91 |
| No attempt | 43 (63.2) | 165 (62.5) |  |
| Had attempt | 25 (36.8) | 99 (37.5) |  |
| Intention to quit |  |  | **0.050** |
| Within 7 days | 39 (54.2) | 106 (41.9) |  |
| Within 30 days | 26 (36.1) | 87 (34.4) |  |
| Within 60 days | 1 (1.4) | 20 (7.9) |  |
| Undecided | 6 (8.3) | 40 (15.8) |  |
| Perceptions of quitting (Score: 0-10) ^b^  [median (IQR)] |  |  |  |
| Perceived importance of quitting | 7 (5; 10) | 7 (5; 10) | 0.46 |
| Perceived difficulty of quitting | 7 (5; 10) | 7 (5; 9) | 0.99 |
| Perceived confidence of quitting | 5 (5; 6) | 5 (5; 6) | 0.82 |

Note

^a^ Being an employer, employee, or self-employed was regarded as economically active; being a student, housekeeper, retired or unemployed was regarded as economically inactive.

^b^ Scored 0-10, higher score indicates stronger perceptions.

P values from chi-square tests, Fisher’s exact test and Wilcoxon rank-sum tests.

IQR, inter-quartile range; median and IQR for continuous variables that are not normally distributed.

Table S2. Sensitivity analyses of smoking abstinence based on complete cases and multiple imputation

|  | Complete cases | | Multiple imputation | |
| --- | --- | --- | --- | --- |
|  | Crude RR  (95% CI) | Adjusted RR ^a^  (95% CI) | Crude RR  (95% CI) | Adjusted RR ^a^  (95% CI) |
| Validated abstinence |  |  |  |  |
| 6-month | 1.24 (0.57-2.68) | 1.28 (0.59-2.76) | 1.21 (0.56-2.59) | 1.24 (0.58-2.64) |
| 12-month | 0.89 (0.49-1.61) | 0.90 (0.50-1.63) | 0.80 (0.45-1.44) | 0.84 (0.47-1.49) |
| Self-reported 7-day PPA |  |  |  |  |
| 6-month | 1.07 (0.67-1.72) | 1.13 (0.72-1.80) | 1.06 (0.67-1.67) | 1.11 (0.71-1.75) |
| 12-month | 0.90 (0.63-1.29) | 0.92 (0.65-1.30) | 0.84 (0.58-1.21) | 0.88 (0.62-1.27) |
| Self-reported 24-week continuous abstinence |  |  |  |  |
| 6-month | 3.57 (1.01-12.65) * | 3.65 (1.04-12.75) * | 3.56 (0.98-12.92) | 3.62 (1.00-13.11) * |
| 12-month | 1.03 (0.58-1.82) | 1.06 (0.61-1.84) | 0.94 (0.53-1.67) | 1.02 (0.58-1.79) |

Note

^a^ Adjusted for previous quit attempt.

Complete case analysis: participants with missing outcomes were excluded.

Multiple imputation analysis: missing data were handled by multiple imputation by chained equations; 50 imputed datasets were created; the regression results were pooled according to Rubin’s rule.

RR: risk ratio; CI: confidence interval; PPA: point prevalence abstinence

* P<0.05

Table S3. Post-hoc analyses of self-reported abstinence outcomes at 3-month

|  | Intervention  N=350 | Control  N=350 | Crude RR  (95% CI) | Adjusted RR ^a^  (95% CI) |
| --- | --- | --- | --- | --- |
|  | n (%) | n (%) |  |  |
| Self-reported 7-day PPA | 35 (10.0) | 25 (7.1) | 1.40 (0.86-2.29) | 1.51 (0.93-2.45) |
| Smoking reduction by at least 50% of baseline ^b^ | 102 (29.1) | 89 (25.4) | 1.15 (0.90-1.46) | 1.17 (0.92-1.49) |
| Any quit attempt since intervention initiation | 35 (10.0) | 25 (7.1) | 1.40 (0.86-2.29) | 1.51 (0.93-2.45) |
| Any use of SC service since intervention initiation | 50 (14.3) | 48 (13.7) | 1.04 (0.72-1.50) | 1.04 (0.72-1.51) |

Note

^a^ Adjusted for previous quit attempt.

^b^ Self-reported quitters included.

RR: risk ratio; CI: confidence interval; PPA: point prevalence abstinence; SC: smoking cessation

Table S4. Post-hoc analyses by whether chatting with smoking cessation advisors in intervention group participants (N=350)

|  | Chatted  N=74 | Not chatted  N=276 | Crude RR  (95% CI) | Adjusted RR ^a^  (95% CI) |
| --- | --- | --- | --- | --- |
|  | n (%) | n (%) |  |  |
| Validated abstinence |  |  |  |  |
| 6-month | 7 (9.5) | 7 (2.5) | 3.73 (1.35-10.31) * | 3.29 (1.13-9.63) * |
| 12-month | 7 (9.5) | 12 (4.3) | 2.18 (0.89-5.34) | 1.73 (0.60-4.99) |
| Self-reported 7-day PPA |  |  |  |  |
| 6-month | 10 (13.5) | 22 (8.0) | 1.70 (0.84-3.42) | 1.83 (0.90-3.73) |
| 12-month | 14 (18.9) | 32 (11.6) | 1.63 (0.92-2.90) | 1.54 (0.78-3.05) |
| Self-reported 24-week continuous abstinence |  |  |  |  |
| 6-month | 5 (6.8) | 6 (2.2) | 3.11 (0.97-9.92) | 2.56 (0.75-8.75) |
| 12-month | 6 (8.1) | 16 (5.8) | 1.40 (0.57-3.45) | 1.23 (0.43-3.50) |
| Smoking reduction by at least 50% of baseline ^b^ |  |  |  |  |
| 6-month | 31 (41.9) | 69 (25.0) | 1.68 (1.20-2.35) ** | 1.75 (1.21-2.55) ** |
| 12-month | 36 (48.6) | 75 (27.2) | 1.79 (1.32-2.43) *** | 1.92 (1.36-2.71) *** |
| Any quit attempt since intervention initiation |  |  |  |  |
| 6-month | 14 (18.9) | 35 (12.7) | 1.49 (0.85-2.62) | 1.37 (0.74-2.54) |
| 12-month | 22 (29.7) | 50 (18.1) | 1.64 (1.07-2.53) * | 1.56 (0.96-2.52) |
| Any use of SC service since intervention initiation |  |  |  |  |
| 6-month | 33 (44.6) | 57 (20.7) | 2.16 (1.53-3.05) *** | 1.66 (1.14-2.43) ** |
| 12-month | 43 (58.1) | 80 (29.0) | 2.00 (1.53-2.62) *** | 1.67 (1.26-2.23) *** |

Note

^a^ Adjusted for intention to quit and children living together at baseline because of their significant difference between participants who chatted or did not chat with SC advisors (Table S1).

^b^ Self-reported quitters included.

RR: risk ratio; CI: confidence interval; PPA: point prevalence abstinence; SC: smoking cessation

* P<0.05; ** P<0.01; *** P<0.001

Table S5. Perceived helpfulness of messages (including chatting for intervention group participants) at 6-month

|  | Median (IQR) | | | P value |
| --- | --- | --- | --- | --- |
|  | Intervention | | Control |  |
|  | Engaged in chatting | Not engaged in chatting |  |  |
|  | N=74 | N=92 | N=150 |  |
| Perceived helpfulness for increasing quitting motivation | 7 (5; 8) | 5 (4; 7) | 5 (2; 7) | **0.006** |
| Perceived helpfulness for increasing quitting confidence | 6 (5; 8) | 5 (2; 7) | 5 (0; 7) | **0.006** |
| Perceived overall helpfulness for quitting | 6 (5; 8) | 5 (5; 7) | 5 (4; 7) | 0.23 |

Note

Scored 0-10, higher score indicates greater perceived helpfulness.

Participants who did not respond to corresponding questions on perceived helpfulness of messages were excluded.

P values for 3-group comparison from Kruskal-Wallis tests.

IQR, inter-quartile range; median and IQR for continuous variables that are not normally distributed.
